# Assessment of the Adequacy of Thyroid Hormone Replacement Therapy in Hypothyroidism

**DOI:** 10.3389/fendo.2019.00631

**Published:** 2019-09-20

**Authors:** Matvey Brokhin, Sara Danzi, Irwin Klein

**Affiliations:** ^1^Private Practice, Brooklyn, NY, United States; ^2^Department of Biological Sciences and Geology, Queensborough Community College, City University of New York, Bayside, NY, United States; ^3^Department of Medicine, NYU School of Medicine, New York, NY, United States

**Keywords:** symptom scale, T4, T3, treatment, diagnosis of hypothyroidism

## Abstract

**Background:** Recent studies identify a significant number of treated hypothyroid patients who express dissatisfaction with their therapy. At present there are sufficient measures of thyroid function to enable the clinician to establish a diagnosis of thyroid disease with a high degree of sensitivity and specificity. The purpose of this study was to quantitate the use of a new and novel assessment of clinically relevant hypothyroid symptoms in the management of patients with thyroid disease and to identify a tool that could help clinicians to assess adequacy of LT_4_ treatment.

**Methodology:** Unselected outpatients of the Thyroid Clinic of the North Shore University Hospital at Manhasset completed a questionnaire asking them to rate their physical symptoms related to thyroid disease as part of their standard care. This questionnaire consisted of 10 signs and symptoms. The questionnaire was collected from 198 control subjects, 241 subjects with primary hypothyroidism (under treatment), 113 euthyroid subjects (benign nodular thyroid disease), 73 previously hyperthyroid subjects (previously treated), and 27 subjects with thyroid cancer. A repeat questionnaire was obtained from 48 subjects with primary hypothyroidism (20%), 19 euthyroid subjects (17%), and 17 subjects previously hyperthyroid (23%).

**Data Analysis:** The mean score for the sum of the signs and symptoms in the primary hypothyroid group with no medication change was 9.62 ± 1.29 for the initial questionnaire, and 10.04 ± 1.32 for the follow up questionnaire (not significant). For the primary hypothyroid patients requiring a medication change, at the time of the initial questionnaire the mean serum TSH was 12.86 ± 2.75 mcU/ml. Concurrently with the normalization of TSH, a statistically significant improvement in the sum of signs and symptoms mean score for this group was noted (16.32 ± 1.93 initial vs. 10.32 ± 1.46 after treatment to normalize TSH).

**Conclusion:** The proposed newly devised hypothyroid scale correctly identified subjects with TSH elevation and clinical/subclinical hypothyroidism based on their clinical signs and symptoms. In this particular subset of patients, the hypothyroid symptom scale showed a statistically significant improvement in the sum of the signs and symptoms with the normalization of the subjects' thyroid function.

## Introduction

There are a number of thyroid function tests (TFTs), which enable the clinician to establish a diagnosis of thyroid disease with a high degree of sensitivity and specificity. The application of these tests, particularly the TSH, makes the diagnosis of overt thyroid dysfunction fairly straightforward in most cases. There are however notable exceptions in which these laboratory tests may not be a reliable measure of thyroid hormone action in individual patients (1). These conditions include, but are not limited to, secondary hyper and hypothyroidism, subclinical forms of hypothyroidism and hyperthyroidism, thyroid hormone resistance, alterations in thyroid hormone binding by a variety of serum carrier proteins, interactions with different medications such as lithium, amiodarone, and iodine, as well as nonthyroidal (low T_3_) illnesses (2–8). In the assessment of these patients, it would be useful to have an additional quantitative clinical measure that both directly and indirectly reflects the cellular action of thyroid hormone and could be used clinically to aid in diagnosis and treatment (1, 8–10). The recent interest in T_4_/T_3_ combination therapy in patients treated with L-T_4_ monotherapy calls out for a quantitative measure of well-recognized hypothyroid symptoms and the response of these symptoms to individualized treatments. Hypothyroid symptom scales have been previously developed and modified over the years to primarily aid in the diagnosis of hypothyroidism (11, 12) to target those patients who would likely be candidates for further testing, but none to date have been designed to assess the adequacy of treatment in patients currently on thyroid hormone replacement with persistent symptoms and to potentially guide novel therapeutic regimens.

Fifty years ago, before the development of adequate and reliable TFTs, Billewicz et al. (11) described a diagnostic index that assessed the presence or absence of various signs and symptoms of hypothyroidism for the purpose of establishing a diagnosis of hypothyroidism. With the development of newer, more reliable TFTs, in 1997 Zulewski et al. (12) revised the hypothyroid signs and symptoms clinical score for individual assessment of the severity of thyroid failure. It was designed based on signs and symptoms originally chosen by Billewicz and a scoring range was determined in patients with untreated overt hypothyroidism and compared to patients with normal thyroid function. Scores were based on the presence (1) or absence (0) of hypothyroid signs and symptoms. Ranges were determined based on average scores in patients and controls and the frequency of these symptoms in the same populations. In addition, a correction factor (+1) was added to a patient's score when his (her) age was <55 years. The diagnostic range for this clinical score was established as ≤ 2 = euthyroid; 3–5 = intermediate; >5 = overt hypothyroid. In overt hypothyroidism the average score was found to correlate with ankle reflex relaxation time, total cholesterol, and creatine kinase. However, while the score correlated with fT_4_ and fT_3_, it did not correlate with TSH, the gold standard for thyroid function testing. TSH and fT_4_ correlated with scores in the middle range. The study concluded that the classical signs and symptoms of hypothyroidism were only present in patients with severe overt hypothyroidism with low serum T_3_ levels, but were minimal or absent in patients with normal T_3_, despite low fT_4_ or in patients with subclinical hypothyroidism (12). Therefore, it seems that T_3_ levels were the most reliable predictor of hypothyroid signs and symptoms. Another important feature of both the Billewicz and Zulewski diagnostic tools is that the scoring is accomplished by physicians who function as examiners, and not by a questionnaire administered to the patient.

It has further been demonstrated that physical examination alone in the diagnosis of hypothyroidism cannot confirm or rule out hypothyroidism without including TFTs (13). To assess the significance of clinical vs. biochemical assessment of hypothyroidism in patients to optimize L-T_4_ dosing, the Billewicz scale was used and compared to biochemical measures (14). Of almost 400 subjects found to be biochemically hypothyroid, <1 fourth could be classified as hypothyroid based on the Billewicz score.

Contrary to the Billewicz and Zulewski scales, in 1997, Canaris et al. (15) examined a group of hypothyroid patients and compared them to matched controls by self -administered questionnaire. In this study, only newly diagnosed patients with a TSH above 20 μU/mL and decreased T_4_ were eligible to participate. The number and percentage of positive symptoms were determined for each patient. Symptoms were scored as present or absent (positive or negative). Using a scale of 1–5, only severe ratings of 4 or 5 were counted as present, ratings of 1–3 were considered absent. Patients were also asked whether the symptom or sign had changed within the past year. For hypothyroid patients, the number of conventional hypothyroid symptoms reported in that group was directly, albeit weakly, correlated to TSH, with a stronger association when more symptoms were reported. Likelihood ratios for the scoring ranges were determined to help assess whether to test for thyroid disease.

Each of the studies described was designed for the purpose of diagnosis of hypothyroidism to aid in deciding whether TFTs are warranted. We have devised a novel hypothyroid symptom scale to assess the adequacy of thyroid hormone replacement therapy. The symptoms were chosen based on those commonly reported to be associated with hypothyroid symptoms that could be self assessed by the patient (10–16). Using this tool, we have identified persistently symptomatic hypothyroid patients who subsequent to appropriate changes in levothyroxine dosages then showed improvement.

## Methodology

### Patient Acquisition

Four hundred fifty-four unselected outpatients attending the Thyroid Clinic of the North Shore University Hospital at Manhasset, and 198 control women undergoing routine outpatient mammography were randomly asked to complete a questionnaire asking them to rate their physical symptoms related to thyroid disease. Follow up questionnaires were randomly obtained at the standard 3–6 month return visit to the Thyroid Clinic.

### Questionnaire

**Figure 1:**
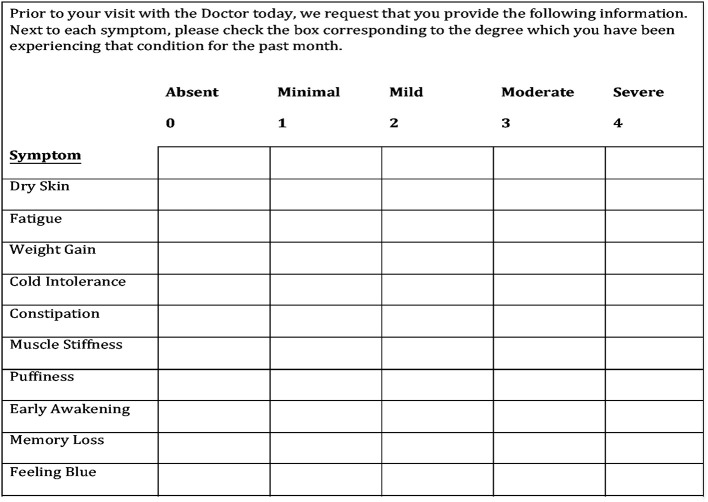
The hypothyroid signs and symptoms questionnaire. The final 10-symptom questionnaire is the result of refining an initial 12-symptom questionnaire after it was determined that two symptoms have no sensitivity or specificity for hypothyroidism.

This questionnaire consisted of 10 signs and symptoms including dry skin, fatigue, weight gain, cold intolerance, constipation, muscle stiffness, puffiness, memory loss, feeling blue, and dizziness. The severity of each sign and symptom was rated on a scale from 0 to 4 points: absent (0), minimal (1), mild (2), moderate (3), and severe (4). The item scores were totaled to obtain the overall signs and symptoms score ranging from 0 to 40 points (Figure 1).

### Data Analysis

Data was collected from 652 patients from the department's patient population. The project was reviewed by the North Shore -LIJ IRB and found to be exempt. Patient and subject consents were not required. The data elements collected included patient age, patient ratings of physical symptoms and thyroid function measurements. For the controls, only age and physical symptoms were collected. Thyroid function measurements included TSH, T_4_, and T_3_ hormone levels. Patients were divided into groups based on their thyroid disease diagnosis for data analysis. The questionnaire was collected from 241 subjects with primary hypothyroidism (under treatment), 113 euthyroid subjects (benign nodular thyroid disease), 73 previously hyperthyroid subjects, and 27 subjects with thyroid cancer in addition to 198 control subjects with no known thyroid disease. Patients with hyperthyroidism were treated with either ^131^I or methimazole and thus became hypothyroid requiring replacement therapy. Thyroid cancer patients who have been thyroidectomized were also treated with thyroid hormone replacement therapy. A repeat questionnaire was obtained from 48 subjects with primary hypothyroidism (20%), 19 euthyroid subjects (17%), and 17 subjects who were previously hyperthyroid (23%) (Table 1). Data are expressed as the mean ± SE. Statistical analysis of the data obtained was done using SPSS 12.0.1 and Excel software.

**Table 1:** Number of questionnaires obtained for each group.

	**Initial**	**Follow-up**	**%**
Control	198	0	–
Primary hypothyroid	241	48	20
Previously hyperthyroid	73	17	23
Thyroid cancer	27	0	0
Euthyroid	113	19	17
Total	652	84	–

## Results

**Figure 2:**
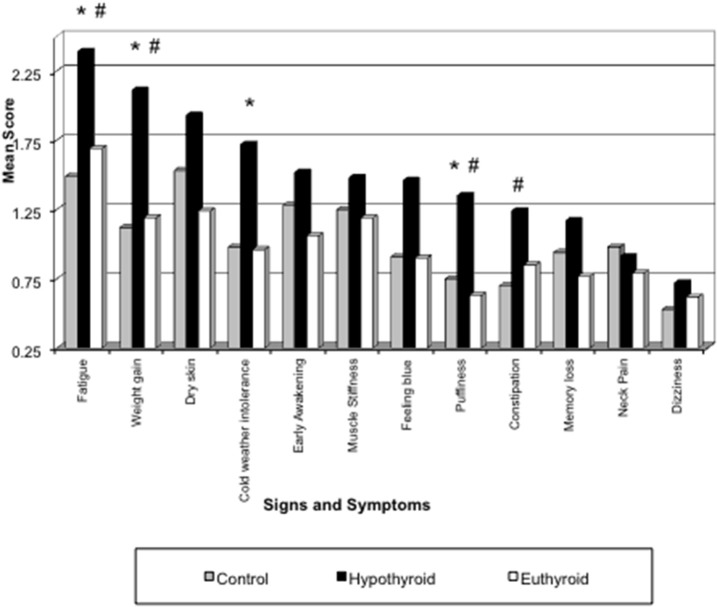
Distribution of signs and symptoms among the study groups. *p ≤ 0.05 for Hypothyroid vs. Euthyroid; ^#^*p* ≤ 0.05 for Hypothyroid vs. Control.

Table 2 summarizes our findings of all the study groups in this project for the sum of signs and symptoms, TSH, T_4_, T_3_, and the study subjects' age. The primary hypothyroid group had the highest ratings of symptoms as compared to other patients or controls despite treatment with levothyroxine sodium. Thyroid function tests (TSH, T_4_, and T_3_) also corresponded to the study groups' diagnoses. Thyroid function tests were not determined in the control study population. The mean age of all study participants was similar except for the thyroid cancer group. The mean of all symptoms in hypothyroid patients was significantly greater (13.6 ± 0.3) compared to all other groups (Table 2). The distribution of signs and symptoms among the study groups is shown in Figure 2. Significant differences (*p* ≤ 0.05) between hypothyroid and euthyroid subjects were found for fatigue, weight gain, cold weather intolerance, and puffiness. Significant differences (*p* ≤ 0.05) between hypothyroid and controls subjects were found for fatigue, weight gain, puffiness, and constipation.

**Table 2:** Sum of signs and symptoms, TSH, T_4_, T_3_ for each group.

**Group Diagnosis**		**Sum of signs and symptoms**	**TSH (mcU/mL)**	**T_**4**_ (mcg/dL)**	**T_**3**_ (ng/dL)**	**Age (years)**
1–Control	Mean ± SE	10.15 ± 0.56	Not Determined			55.83 ± 0.93
	N	198		198		
2–Primary Hypothyroid	Mean ± SE	13.60 ± 0.53	2.16 ± 0.37	9.38 ± 0.19	112.5 ± 3.30	52.16 ± 0.88
	N	241	215	172	85	241
3–Previously Hyperthyroid	Mean ± SE	9.47 ±0.92	0.44 ± 0.12	9.46 ± 0.65	169.1 ± 15.47	52.34 ± 1.92
	N	73	50	29	28	73
4–Thyroid cancer	Mean ± SE	10.93 ± 1.59	0.22 ± 0.11	11.09 ± 1.23	135.4 ± 19.37	45.48 ± 2.51
	N	27	20	19	5	27
5–Euthyroid	Mean ± SE	9.76 ± 0.70	1.43 ± 0.09	8.45 ± 0.28	136.4 ± 6.58	51.58 ± 1.46
	N	113	107	60	35	113

Within the primary hypothyroid group of subjects, 48 follow up questionnaires were obtained out of the original 241 study participants. The 48 follow up questionnaires included 22 study subjects who required increased thyroid medication during the study period and 26 study subjects who did not. The analyzed data also included serum TSH, T_4_, and T_3_ levels, which were obtained at the same time as the initial and follow up questionnaires.

The mean score for the sum of the signs and symptoms in the primary hypothyroid group with no medication change was 9.62 ± 1.29 for the initial questionnaire, and 10.04 ± 1.32 for the follow up questionnaire (*p* = 0.517) (Table 3).

**Table 3:** Test-retest data analysis for the primary hypothyroid group.

**A**				**B**			
**Primary Hypothyroid without Rx change**				**Primary Hypothyroid with Rx increase**			
**Test - 1, Retest - 2**	**Mean** **±** **SE**	**N**	**Sig**.	**Test - 1, Retest - 2**	**Mean** **±** **SE**	**N**	**Sig**.
**Sum of signs and symptoms**				**Sum of signs and symptoms**			
1	9.62 ± 1.29	26	NS	1	16.32 ± 1.93	22	0.0002
2	10.04 ± 1.32	26		2	10.32 ± 1.46	22	
**TSH (mcU/mL)**				**TSH (mcU/mL)**			
1	1.23 ± 0.19	26	NS	1	12.86 ± 2.75	22	0.001
2	1.76 ± 0.48	5		2	1.89 ± 0.35	21	
**T**_**4**_ **(mcg/dL)**				**T**_**4**_ **(mcg/dL)**			
1	9.25 ± 0.39	26	NS	1	7.20 ± 0.61	22	0.01
2	9.20 ± 1.45	4		2	9.76 ± 0.55	20	
**T**_**3**_ **(ng/dL)**				**T**_**3**_ **(ng/dL)**			
1	108.6 ± 5.9	10	NS	1	99.0 ± 11.94	12	0.01
2	136.0	1		2	137.75 ± 7.84	12	
**Levothyroxine sodium dose (mcg)**				**Levothyroxine sodium dose (mcg)**			
1	95.29 ± 8.01	26	NS	1	72.37 ± 10.53	22	0.034
2	95.29 ± 8.01	26		2	100.37 ± 7.67	22	
**Age (years)**				**Age (years)**			
1	51.40 ± 2.76	26	NS	1	49.60 ± 2.75	22	NS
2	51.53 ± 2.76	26		2	49.92 ± 2.76	22	

For the primary hypothyroid patients requiring a medication change, at the time of the initial questionnaire the mean serum TSH was 12.86 ± 2.75 mcU/ml. With an average increase in levothyroxine from 72.37 ± 10.53 mcg to 100.37 ± 7.67 mcg between the initial and follow up points of the study, a statistically significant decrease of the mean TSH to 1.89 ± 0.35 was observed (*p* = 0.001). Concurrently with the normalization of TSH, a statistically significant improvement in the sum of signs and symptoms mean score for this group was noted (Table 3) (*p* = 0.0002). As expected, T_4_ and T_3_ levels increased, but remained within the normal range after the increase of levothyroxine sodium (*p* = 0.003 and 0.017, respectively).

## Discussion

The results of our study demonstrated reproducibility and validity of this newly proposed hypothyroid signs and symptoms scale. There was no statistically significant change in the sum of the signs and symptoms in the primary hypothyroid group with no medication change between the initial and follow up visits, suggesting this novel scale's reproducibility. Also, the proposed hypothyroid signs and symptoms scale correctly identified patients with untreated or under treated primary hypothyroidism with the total mean score of 16.74 as compared to the euthyroid and control groups with the total mean scores of 11.31 and 12.40, respectively, suggesting this scale's validity.

Similarly to the results of Canaris et al. (15), our data showed that the symptoms associated with hypothyroidism were predictive of abnormal serum TSH, and the study participants with higher symptoms scores on the hypothyroid scale had significantly higher TSH values. Similarly to the results of Zulewski et al. (12), our hypothyroid scale showed a significantly higher signs and symptoms scores in the untreated or under treated hypothyroid group of patients as compared to the “baseline” score of the control, euthyroid, and adequately thyroxine—replaced hypothyroid study groups.

McAninch et al. (16) conducted a retrospective study that included 99 studies of hypothyroid patients treated with T_4_ monotherapy. Previous observations suggested that monotherapy with T_4_ may not be adequate because patients complained of persistent symptoms. Results demonstrated that in patients with T_4_ monotherapy and normalized serum TSH, not all systemic markers of thyroid hormone signaling were normalized, including serum LDL and total cholesterol. The failure to restore a euthyroid state with levothyroxine monotherapy may explain the ~15% dissatisfaction with hypothyroid patients treated with T_4_ alone. This is supported by Peterson et al. (17) who conducted an online survey of hypothyroid patients to determine their level of satisfaction with their current therapy or their physician. Higher satisfaction was reported by patients receiving desiccated thyroid extract followed by patients receiving combination T_4_ plus T_3_. The lowest satisfaction level was found in patients on T_4_ monotherapy. The study does not distinguish between physician and therapy dissatisfaction, but one can assume they are related.

A recent study reported on quality of life measures in hypothyroid patients using the Thyroid Patient-Reported Outcome (ThyPRO-39) questionnaire. Patients were on T_4_ monotherapy but experiencing persistent symptoms (18). Patients switched to combination LT_4_/LT_3_ combination therapy showed an improvement in quality of life measures, which was not associated with a change in TSH.

In preclinical studies, Escobar-Morreale et al. (19) demonstrated that the infusion of T_4_ alone to hypothyroid rats, at any dose, cannot normalize TSH, T_4_, and T_3_ in the blood, or in all tissues of the hypothyroid animal. In order to ensure normal T_3_ levels in all tissues supraphysiological plasma levels of T_4_ resulted. The minimal dose of T_4_ that resulted in normal plasma T_4_ and T_3_ was insufficient to normalize the concentration of T_3_ in most tissues analyzed including heart, lung, liver and kidney. Results also demonstrated differential uptake of circulating T_4_. This implies that in humans current replacement therapy with T_4_ alone would not be adequate to render all tissues euthyroid. In a second study by the same group (20) infusion of hypothyroid rats with T_4_ alone or T_4_ in combination with T_3_ (at three different doses) demonstrated that tissue euthyroidism is only possible when T_4_ is infused together with T_3_. In all treatment groups, plasma T_4_ was normalized but TSH and T_3_ in plasma and T_3_ in most tissues were only normalized with the combination of T_4_ plus T_3_.

In a clinical study by Celi et al. (21) 14 hypothyroid patients received either T_4_ monotherapy or T_3_ administered three times a day at doses that produced equivalent normalization of serum TSH. No difference was noted in TSH between groups. Results demonstrated that T_3_ treatment resulted in significant weight loss and a more favorable lipid profile (decreased total cholesterol and LDL cholesterol) when compared to T_4_ treatment, implying adequate tissue levels of serum T_3_ in these patients. T_3_ treatment produced no differences in cardiovascular function (heart rate, blood pressure, or exercise tolerance), HDLs, or in insulin sensitivity when compared to T_4_.

An easy to use hypothyroid symptom scale that identifies those patients with persistent symptoms is a critical component for addressing the needs of the 15% of patients who are not “satisfied” on their current therapy. In the case of suspected inadequate thyroid hormone replacement, the clinician may opt for LT4 dose increase, change in LT4 formulation or the addition of liothyronine (22). The proposed hypothyroid scale correctly identified the subjects with TSH elevation and clinical/subclinical hypothyroidism based on their clinical signs and symptoms. In this particular subset of patients, the hypothyroid symptom scale showed a statistically significant improvement in the sum of the signs and symptoms with the normalization of the subjects' thyroid function. At the same time, the sum of the signs and symptoms of patients with no change in medication remained unchanged, suggesting a reproducibility of this hypothyroid scale. This scale provides the clinician with an easily applied clinical tool to assess the adequacy of treatment in hypothyroid patients.

## Data Availability

The datasets generated for this study are available on request to the corresponding author.

## Ethics Statement

Reviewed by the North-Shore Hospital IRB in Manhasset, NY. Study was found to be EXEMPT as no identifying information was obtained.

## Author Contributions

MB and IK: data collection, analysis, and manuscript preparation. SD: manuscript preparation and data analysis.

### Conflict of Interest Statement

The authors declare that the research was conducted in the absence of any commercial or financial relationships that could be construed as a potential conflict of interest.
